# Are cystic fibrosis mutation carriers a potentially highly vulnerable group to COVID‐19?

**DOI:** 10.1111/jcmm.15941

**Published:** 2020-10-03

**Authors:** Panagiotis Sarantis, Evangelos Koustas, Athanasios G. Papavassiliou, Michalis V. Karamouzis

**Affiliations:** ^1^ Molecular Oncology Unit Department of Biological Chemistry Medical School National and Kapodistrian University of Athens Athens Greece

**Keywords:** ACE‐2, COVID‐19, CTFR, cystic fibrosis, SARS‐CoV‐2

## Abstract

Undoubtedly, the new SARS‐CoV‐2 virus poses a grave health threat, plaguing the health and socio‐economic sectors. COVID‐19 disease must be treated quickly and effectively as soon as possible. The main axes in this direction are establishing vaccines, drugs, diagnostic tests, as well as identifying the most vulnerable groups. Probably, there is a correlation between COVID‐19 and cystic fibrosis. Our interest is focused on cystic fibrosis carriers that, due to limited tests, remain undetectable. There is an activation of the inflammatory response in the carriers, as well as in cystic fibrosis patients. First of all, a striking similarity lies between the inflammatory response in COVID‐19 and cystic fibrosis carriers. Notably, ACE‐2 plays the same role in both cases and a similar geographical distribution is observed in both diseases. In conclusion, we suggest that cystic fibrosis mutation carriers are potential members of a certain vulnerable group and the detection of such mutations in the population might be vital for the prevention of SARS‐CoV‐2 virus, and more specifically to limit its serious complications.

## 1 CYSTIC FIBROSIS AND *CTFR* GENE

Cystic fibrosis (CF) is an autosomal recessive disorder caused by mutations in the cystic fibrosis transmembrane regulator (*CFTR*) gene, located on the long arm of chromosome 7. The first clinical description of the syndrome was in 1939, and the responsible gene was successfully cloned in 1989.^1^

The CFTR gene encodes an adenosine triphosphate (ATP)‐binding cassette (ABC) transporter of the cell membrane. The term CFTR‐related disorders (CFTR‐RD) describe a subgroup of patients with marked CFTR dysfunction, but they do not complete the CF diagnosis criteria. This term involves three discrete clinical entities: congenital bilateral absence of the vas deferens, acute recurrent or chronic pancreatitis and disseminated bronchiectasis.^2^

One of the most common mutations in the CFTR gene is functional significance 9 (ΔF508 or F508del) and is present in approximately 85% of CF patients worldwide. F508del caused by a phenylalanine deletion at position 508 on chromosome 7, and it accounts for over two‐thirds of all detected mutations in northern Caucasian populations.^3^

## 2 INFLAMMATION AND CF

It is widely known that in the presence of ΔF508‐CFTR mutation, NFκB appears to be constitutively activated. This leads to IL‐8 depended chronic neutrophilic lung disease. New evidence supports the hypothesis that the imbalance of inflammation response appears to be an intrinsic component of the CF and airway, and lung inflammation may occur in absence or infection. In addition, lung epithelial cells, with mtCFTR, secrete pro‐inflammatory cytokines and enhance the activation of NFκB.^4^

The mechanism by which mt CFTR leads to abnormalities of the NFκB inflammation pathway is still ambiguous. TRADD (tumour necrosis factor receptor type 1‐associated DEATH domain protein) is the main c signalling intermediate component between TNF‐α and NF‐κB.^5^

**Figure 1:**
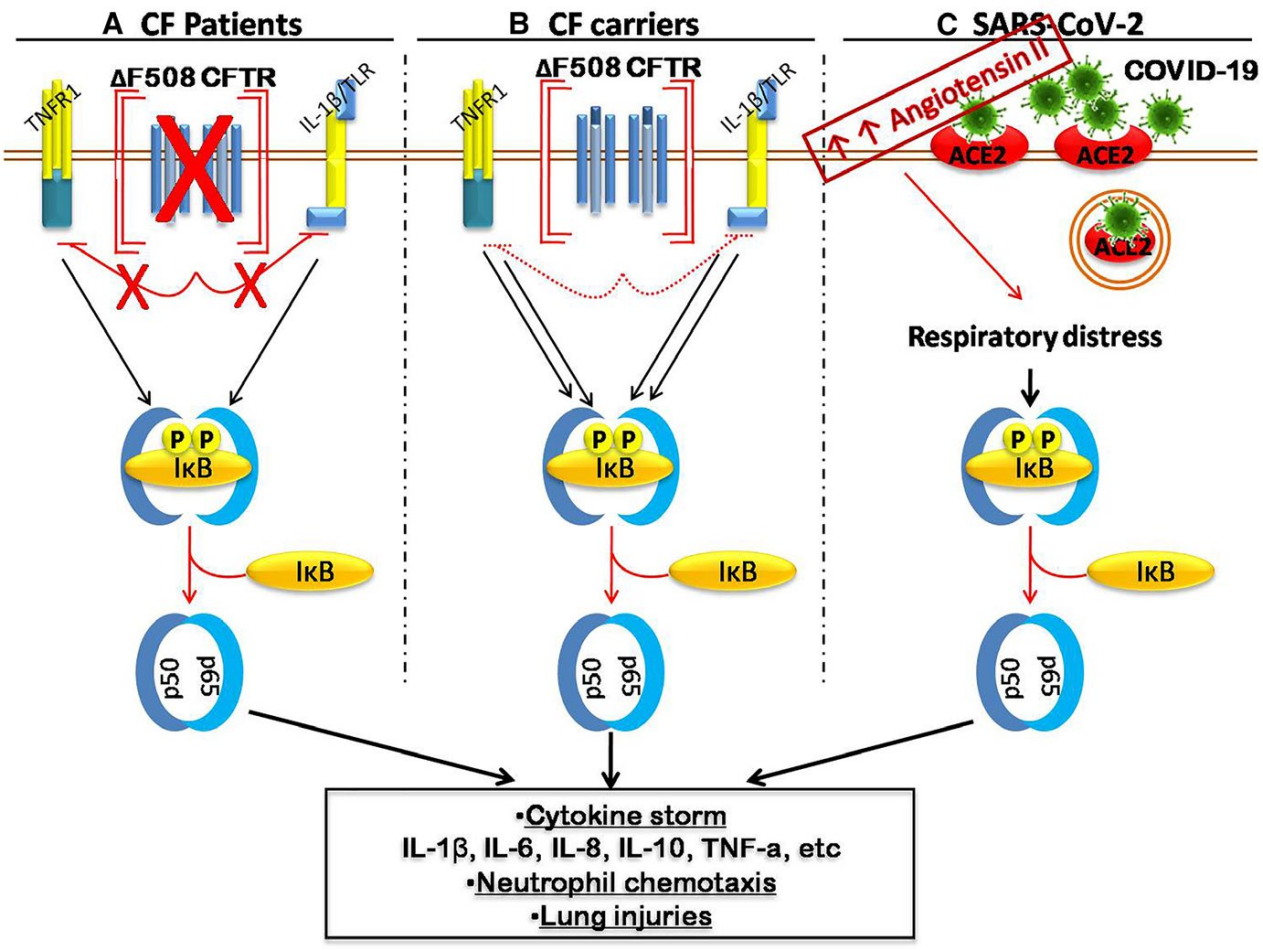
The sensitivity of cystic fibrosis transmembrane regulator (CFTR) carriers to COVID‐19 inflammatory response. The absence of functional CFTR on the surface of the airway cells mediates the inflammatory response in CF that initiates a chronic pro‐inflammatory response through NFκB (A). In the presence of ΔF508‐CFTR on the cell surface of CF heterozygotes is a critical mediator of this hyper‐inflammatory immune response. Furthermore, in the presence of misfolded CFTR protein may also trigger the NFκB inflammation signalling pathway (B). The endocytosis of the ACE‐2 receptor after COVID‐19 binding leads to the accumulation of Angiotensin II and, therefore, respiratory distress and lung injuries through NF‐kB signalling (C). Consequently, heterozygotes of CFTR are more sensitive to cytokine storms mediated by NF‐kB, and the severity of SARS‐CoV‐2 is higher on this population

In CF cases, TLR‐2,3 and 4 appear to be activated, and therefore, an acute innate immune response is triggered through NF‐κB‐depended transcription of inflammatory cytokines genes.^6^ Therefore, NF‐κB translocates to the nucleus and triggers the transcription of pro‐inflammatory genes TNF‐α, IL‐6, IL‐8 and arachidonic acid metabolites.^7^ The cytokine storms in CF patients and carriers of mt CFR are presented in Figure 1.

## 3 CYSTIC FIBROSIS MUTATION CARRIERS ARE AT INCREASED RISK FOR A WIDE RANGE OF CYSTIC FIBROSIS‐RELATED CONDITIONS

Cystic fibrosis carriers are at an elevated risk of developing a broad scale of conditions related to CF in several organs. Carriers have ∼50% as much CFTR anion channel activity as the healthy people have. Unusually acidic airway surface liquid could drive lung disease in CF patients and may elucidate the important risk of lung disease for carriers. Further studies of epithelial Cl^−^ and HCO3^−^ secretion, particularly under stimulated conditions, may yield a better perceptive of how the reduced CFTR may affect or not to the disease in many organs.^8^

Because of the high frequency of CF carriers (about 1 in 25 people of Northern Europe), the probability of respiratory infections and related antibiotic use attributable to the CF carriers could be substantial. Furthermore, the heterozygote state could contribute to persistent respiratory infections. In addition, CF carriers have an elevated risk of asthma than non‐carriers.^9^

## 4 INFLAMMATION AND SARS‐CoV‐2

The vigorous study of SARS‐CoV‐2 infection reveals high plasma levels of a plethora of pro‐inflammatory cytokines, such as MCP, IL1‐*β*, TNF‐*α*, VEGF‐A and IFN*γ*, suggesting the pathogenic role of cytokine storms mediated by overproduction of pro‐inflammatory cytokines related to damage and disease severity in COVID‐19 patients.^10^ Besides, high levels of IL‐1 and IL‐6 are noticed in response to COVID‐19, highlighting that there might be a correlation between cytokine release syndrome and COVID‐19 infection.^11^ Therefore, the unabated overproduction of pro‐inflammatory cytokines (described as a cytokine storm) leads to an increased risk of vascular hyperpermeability, multiorgan failure and eventually death.^12^

Several studies have revealed viral structural and non‐structural proteins that antagonize interferon responses in various steps, including by inhibiting PRR recognition of viral RNA, by preventing PRR axis through TBK1/inhibitor of nuclear factor‐κB kinase subunit‐*ε* (IKK*ε*), TRAF3 and IRF3, by inhibiting downstream interferon signalling through STAT1 and by initiating host mRNA degradation and inhibiting the translation of host protein. Antagonism and alteration of interferon response promote viral replication, which leads to increased release of pyroptosis products and, therefore, further induces inflammatory response.^11^

## 5 ACE CORRELATION WITH CF and SARS‐CoV‐2

ACE‐2 (angiotensin‐converting enzyme 2) is an ectoenzyme that converts angiotensin II to angiotensin (1‐7). The pathogenesis of lung fibrosis involves the down‐regulation of ACE‐2, leading to lung collagen deposition. Additionally, ACE‐2 enzymatic activity is rigorously decreased in both human and experimental animals, lung fibrosis. Besides, in vivo gene silencing of ACE‐2 up‐regulates bleomycin‐induced lung collagen deposition in mice through the amplified levels of ANG II. ANG II has proapoptotic and profibrotic effects in the lungs and other organs, and these dangerous effects of ANG II are reduced by ACE‐2. On the other hand, the administration of ACE‐2 inhibits fibrotic development. ACE‐2 protects against lung fibrogenesis by the restrictive accumulation of ANG II.^13^

ACE‐2 is a cell surface protein that is used by SARS‐CoV‐2 to invade the host cell. The SARS‐CoV‐2 S‐protein is bearing mutations that increase its affinity to human ACE‐2 by ~10‐15‐fold compared to SARS‐CoV S‐protein, making it exceedingly infectious. Once the SARS‐CoV‐2 virus binds to ACE‐2, it prevents ACE‐2 from performing its normal function to regulate ANG II signalling. Consequently, ACE‐2 action is repressed and makes more ANG II available to injure tissues.^14^

The role of ACE‐2 in cytokine storm mediated by COVID‐19 binding is presented in Figure 1.

## 6 GEOGRAPHICAL DISTRIBUTION

**Figure 2:**
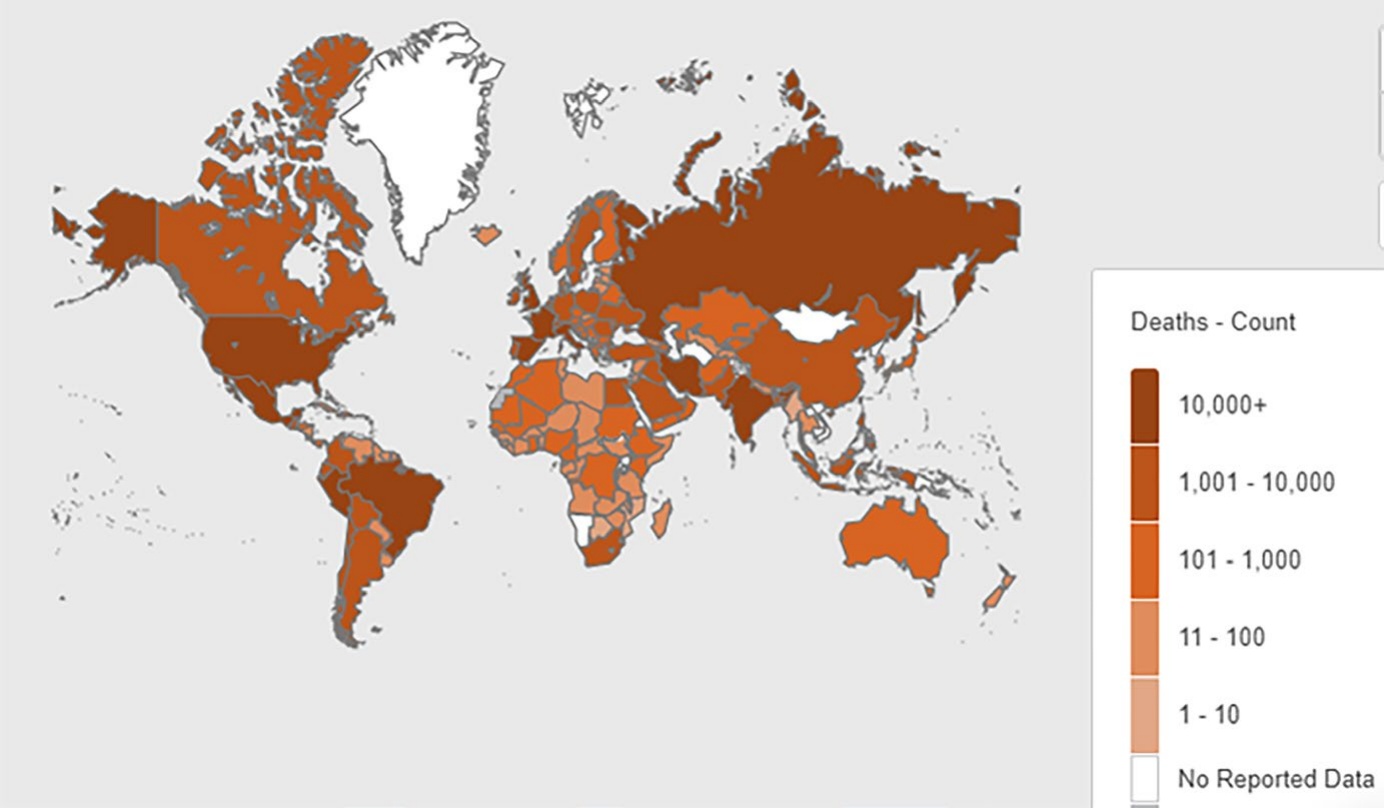
Geographical distribution. The geographical distribution of total deaths caused by COVID‐19 (Map from WHO, on 11 July). In both diseases, Central and Western Europe, as well as North America, have the most significant impact and generally follow the same geographical distribution

Based on the geographical distribution of the CF presented by Bell et al^15^ and the geographical distribution of total deaths due to the COVID‐19 (data from WHO, on 11 July), a similar geographical distribution is observed in both diseases (Figure 2). In both diseases, Central and Western Europe, as well as the USA, have the most significant impact. Russia and Brazil have a severe impact too.

## 7 CONCLUSION OPINION

In the present article, we first describe the typical inflammatory response course and the cytokine cataract between COVID‐19 and cystic fibrosis (patients and carriers). Also, it is seen that there is the direct involvement of ACE‐2 in the pathophysiology of COVID‐19 and cystic fibrosis. Finally, there is an almost identical distribution of cystic fibrosis patients and deaths by COVID‐19 worldwide. These three characteristics are likely to support a correlation between SARS‐CoV‐2 cases and the increased sensitivity of cystic fibrosis mutation carriers.

In conclusion, we suggest the investigation, especially in the most severe COVID‐19 cases, if there is a correlation with cystic fibrosis mutation carriers. If this is proven true, more diagnostic molecular tests for detecting CF mutation carriers in the population will be needed. Such a possible association could create a significant protection net for cystic fibrosis mutation carriers and be vital for the prevention of the SARS‐CoV‐2 virus and its serious complications.

## CONFLICT OF INTEREST

The authors declare no potential conflicts of interest.

## AUTHOR CONTRIBUTIONS

**Panagiotis Sarantis:** Conceptualization (equal); Data curation (equal); Formal analysis (equal); Investigation (equal); Methodology (equal); Resources (equal); Software (equal). **Evangelos Koustas:** Conceptualization (equal); Data curation (equal); Formal analysis (equal); Investigation (equal); Methodology (equal); Project administration (equal); Resources (equal); Software (equal). **Athanasios G Papavassiliou:** Conceptualization (equal); Data curation (equal); Formal analysis (equal); Investigation (equal); Methodology (equal); Project administration (equal); Resources (equal); Supervision (equal); Validation (equal). **Michalis V. Karamouzis:** Conceptualization (equal); Data curation (equal); Formal analysis (equal); Methodology (equal); Project administration (equal); Resources (equal); Supervision (equal).
